# Common predators and factors influencing their abundance in *Anopheles funestus* aquatic habitats in rural south-eastern Tanzania

**DOI:** 10.1371/journal.pone.0287655

**Published:** 2023-06-26

**Authors:** Herieth H. Mahenge, Letus L. Muyaga, Joel D. Nkya, Khamis S. Kifungo, Najat F. Kahamba, Halfan S. Ngowo, Emmanuel W. Kaindoa

**Affiliations:** 1 Environmental Health and Ecological Sciences Department, Ifakara Health Institute, Ifakara, Tanzania; 2 The Nelson Mandela, African Institution of Science and Technology, School of Life Sciences and Bio Engineering, Tengeru, Arusha, United Republic of Tanzania; 3 School of Biodervisty, Animal Health and Comparative Medicine, University of Glasgow, Glasgow, United Kingdom; 4 Wits Research Institute for Malaria, School of Pathology, Faculty of Health Sciences, University of the Witwatersrand and the Centre for Emerging Zoonotic and Parasitic Diseases, National Institute for Communicable Diseases, Johannesburg, South Africa; University of Eldoret, KENYA

## Abstract

**Background:**

The role of larval predators in regulating the *Anopheles funestus* population in various malaria-endemic countries remains relatively unknown. This study aimed to investigate the common predators that co-exist with *Anopheles funestus* group larvae and evaluate factors that influence their abundance in rural south-eastern Tanzania.

**Methods:**

Mosquito larvae and predators were sampled concurrently using standard dipper (350 ml) or 10 L bucket in previously identified aquatic habitats in selected villages in southern Tanzania. Predators and mosquito larvae were identified using standard identification keys. All positive habitats were geo-located and their physical features characterized. Water physicochemical parameters such as dissolved oxygen (DO), pH, electrical conductivity (EC), total dissolved solids (TDS) and temperature were also recorded.

**Results:**

A total of 85 previously identified *An*. *funestus* aquatic habitats in nine villages were sampled for larvae and potential predators. A total of 8,295 predators were sampled. Of these Coenagrionidae 57.7% (n = 4785), Corixidae 12.8% (n = 1,060), Notonectidae 9.9% (n = 822), Aeshnidae 4.9% (n = 405), Amphibian 4.5% (n = 370), Dytiscidae 3.8% (n = 313) were common. A total of 5,260 mosquito larvae were sampled, whereby *Anopheles funestus* group were 60.3% (n = 3,170), *Culex* spp. 24.3% (n = 1,279), *An*. *gambie s*.*l*. 8.3% (n = 438) and other anophelines 7.1% (n = 373). Permanent and aquatic habitats larger than 100m^2^ were positively associated with *An*. *funestus* group larvae (P<0.05) and predator abundance (P<0.05). Habitats with submerged vegetation were negatively associated with *An*. *funestus* group larvae (P<0.05). Only dissolved oxygen (DO) was positively and significantly affect the abundance of *An*. *funestus* group larvae (P<0.05). While predators’ abundance was not impacted by all physicochemical parameters.

**Conclusion:**

Six potential predator families were common in aquatic habitats of *An*. *funestus* group larvae. Additional studies are needed to demonstrate the efficacy of different predators on larval density and adult fitness traits. Interventions leveraging the interaction between mosquitoes and predators can be established to disrupt the transmission potential and survival of the *An*. *funestus* mosquitoes.

## Background

Larval control interventions are part of an integrated malaria vector control approaches such as Indoor Residual Spraying (IRS), Long lasting Insecticides Treated Nets (LLINs), improved diagnostics and treatments [1]. Such interventions are effective, inexpensive and safe to non-target organisms [2–4]. Previous evidence on the use of bacterial larvicides by Derua *et al*., [5], suggested that larviciding should become more important as a vector control tool. However, a greater understanding of the larval biology is essential for effective application. In particular, the role of predators in regulating the population of mosquito larvae remains relatively unstudied.

Many aquatic invertebrate predator species, including Aeshnidae [6], Notonectidae [6] and Dytiscidae [7, 8] coexist with mosquito larvae. Isolating predators and distinguishing them from other organisms can be done by direct observation of their behaviour, visual examination of their midgut contents, molecular methods or electrophoretic methods [9–11]. A single aquatic habitat may contain several species of predators. Such predators have been shown to be effective biocontrol agents against mosquitoes in different larval habitats [6, 8, 12]. For example, the mosquito larval mortality attributed to predators ranges between 54% and 90% depending on the environment, predator species diversity and density [8]. Although, mosquito predators directly, or indirectly, influence mosquito population dynamics [13], their effects on *An*. *funestus* dynamics are not understood. The use of such predators may limit mosquito larval abundance and reduce adult densities [8, 14–16].

Although malaria is transmitted by several *Anopheles* species in Tanzania, *Anopheles funestus* is the primary vector [17–19]. This species is also highly resistant to common insecticides currently used for malaria control, in particular pyrethroids used in insecticide treated nets (ITNs) [20]. Despite a number of studies on the bionomics of these mosquitoes [21–23], and their aquatic habitats [24], the relationship between aquatic predators on *An*. *funestus* larval population remains unknown.

This study investigated the common aquatic predators and factors influencing their abundance in *An*. *funestus* aquatic habitats in south-eastern Tanzania. Specifically, this study aimed to 1) identify types of common predators co-existing with *An*. *funestus* group larvae in a rural part of Tanzania, 2) determine factors which might contributed to the abundance of these predators and 3) quantify the associations between different predator types and *An*. *funestus* group larval abundance.

## Material and methods

### Study area

**Fig 1:**
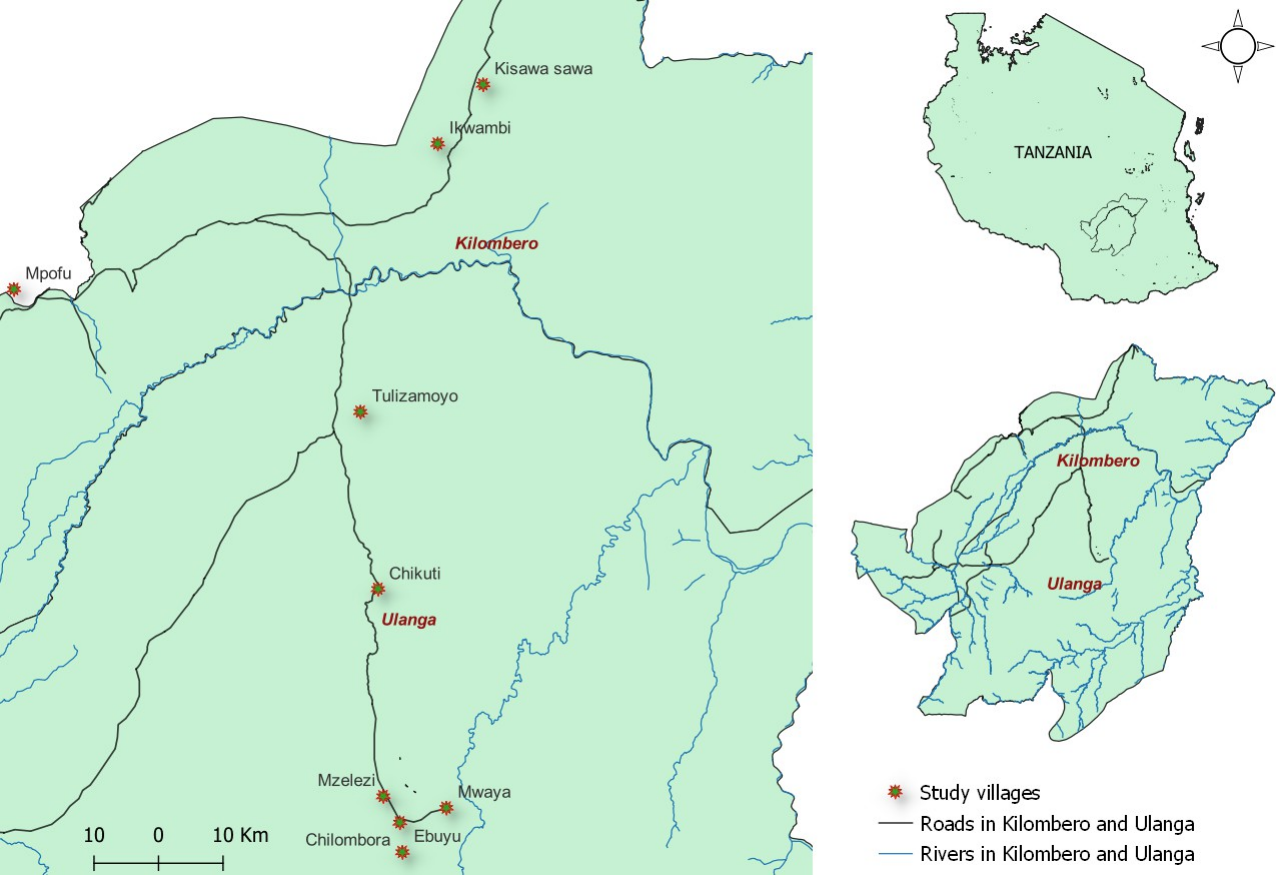
Map of Kilombero and Ulanga districts showing the nine study villages.

A cross-sectional survey was conducted, between March and May 2022, in nine villages in south-eastern Tanzania, namely Chikuti (-8.6028°, 36.7288°), Mzelezi (-8.8934°, 36.7343°), Chirombola (-8.93041°, 36.75753°), Ebuyu (-8.9719°, 36.7608°), Mwaya (-8.91022°, 36.823139°) and Tulizamoyo (-8.35447°, 36.70546°) in Ulanga district and Ikwambi (-7.97927°, 36.81630°), Kisawasawa (-7.89657°, 36.88058°) and Mpofu (-8.17220°, 36.21651°) in Kilombero district (Fig 1). In this area *An*. *funestus* is responsible for more than 85% of overall malaria transmission [17]. The residents in these villages practise extensive rice farming, which creates suitable habitat for mosquito breeding. Common aquatic habitats for *An*. *funestus* in the villages are well known and have been previously characterized [24]. Eighty-five known habitats from the nine villages were sampled for both mosquito larvae and potential predators.

### Sampling and morphological identification of mosquito larvae and aquatic predators

Mosquito larvae and predators were sampled using standard dippers (350 ml) or 10 L buckets, as previously described [8, 15, 24]. A minimum of 3 dips and a maximum of 20 dips were taken depending on the size and depth of the habitat.

In a previous study, mosquito larvae from the same villages were taken to the laboratory in Ifakara, allowed to emerge and eventually identified to species by PCR. Of those identified 53% were *An*. *funestus* s.s. whilst 28% were *An*. *rivulorum* and 12% were *An*. *leesoni* [24]. All three species were found to occupy the same habitats. A similar approach was followed with samples of fourth instar larvae during the present study but identification to species level was not performed. Earlier stage larvae were identified based on their predominant characteristics as done in a previous study and separated into *An*. *funestus* group, *An*. *gambiae* s.l. or *Anopheles* sp. following the identification key by Gilles and Coetzee [25, 26]. Culicines were identified to genera only. Predators were morphologically identified to family level using the keys of the Stroud Water Research Center [27] and Gerber and Gabriel [28]. Mosquito larvae and predators that were sampled by each dipper or bucket were counted and recorded. Additionally, geographical locations of the surveyed habitats were recorded at access points using a hand-held GPS device (Garmin eTrex 20x Handheld GPS Receiver).

### Aquatic habitats characterization

Only positive aquatic habitats for *An*. *funestus* group larvae were sampled for mosquito larvae and predators. Their overall physical characteristics were recorded and physicochemical parameters of the water (pH, temperature, electrical conductivity (EC), total dissolved solids (TDS) were measured using a portable water quality meter (ZJ practical 4 in 1 Water Tester). A Trans Instruments Dissolved Oxygen Meter (HD3030) was used to measure dissolved oxygen (DO), using standard recording procedures. Habitats were classified as being either: swamp, stream, river, rice-field, stream-pool, ground-pool, ditch, spring-fed pool, puddle, hoof-print, man-made wells, brick or sand pit. Water colour was categorized as being clear (transparent and odourless) or coloured (cloudy, not transparent, turbid or with a film of oil).

The source of water was also classified as rainwater or others (non-rainwater). Algal quantities in the habitats were classified as none, moderate, or abundant. Algal type was classified as filamentous, green, blue-green or brown. Water was also classified as being stagnant, slow or fast moving. The land use surrounding the aquatic habitats was classified as scrub, cattle grazing or cultivated field. Shade over the habitats was classified as none, partial or heavy. Habitat size was measured using tape and classified as being less than 100 m^2^ or more than 100 m^2^. Vegetation quantity and vegetation type were also classified as (none, moderate or abundant) and (emergent, or submerged) respectively. Water bodies known to have existed for three months or more were considered to be permanent whilst other collections of water were considered to be ‘temporary’. Water depth was classified as being less than 50 cm or more than 50 cm deep. The distance from aquatic habitats to the nearest houses were estimated visually and classified as being less than 100 m or more than 100 m.

### Statistical analysis

Analysis was done using open source software R version 4.2.1. [29]. Generalised linear mixed effects models (GLMM) using template model builder (TMB) with zero-inflated negative binomial implemented under the *glmmTMB* package [30] were used to (i) assess the associations between water physicochemical parameters and the abundance of aquatic predators ii) assess the associations between water physicochemical parameters and the abundance of *An*. *funestus* group larvae (iii) assess which habitat characteristics contributed to the abundance of predators and *An*. *funestus* group larvae and (iv) assess the impact of each predator family on the abundance of *An*. *funestus* group larvae. All variables (i-iv) were assessed individually and later combined in the final model.

Due to a large number of dips with zero larvae the negative binomial with zero inflated models were used. In all models, the study villages in which the aquatic habitats were identified and habitat ID were used as random terms to capture unexplained variations between villages and habitats. The best fitting models were selected using Akaike Information Criterion (AIC) and results presented as risk ratios (RR) at 95% CI and statistical significance was considered when the P-value < 0.05.

### Ethical considerations

Research proposal was presented to the Nelson Mandela Institute of Science and Technology and approval for this study was obtained from the institutional review board of Ifakara Health Institute (Ref: IHI/IRB/No: 13–2022) and from the Medical Research Coordinating Committee (MRCC) at the National Institute for Medical Research (NIMR) (Ref: NIMR/HQ/R.8a/Vol. IX/3353). The consent of publication this manuscript was obtained from the National Institute for Medical Research (NIMR) (Ref. No: NIMR/HQ/P.12 VOL XXXV/61). Written permission to conduct study was obtained from local leaders in each village whereby the purpose, procedure and benefits of the study were clearly explained. Verbal and written informed consents were obtained from community members who assisted to sample aquatic predators.

## Results

### Distribution of mosquito larvae and their aquatic predators

A total of 85 aquatic habitats that contained *Anopheles funestus* group larvae were identified and characterized. In these habitats, a total of 8,295 predators were sampled. Among all sampled predators, only 7,906 predators were identified belonging to eight families. Of these, Coenagrionidae accounted for 57.7% (n = 4785), Corixidae 12.8% (n = 1,060), Notonectidae 9.9% (n = 822), Aeshnidae 4.9% (n = 405), Amphibian 4.5% (n = 370), Dytiscidae 3.8% (n = 313), Belostomatidae 1.2% (n = 103) and Nepidae 0.6% (n = 48) (Table 2). Three hundred and eighty-nine (4.6% of the total invertebrates) were not identified due to lack of an appropriate key (Table 2). A total of 5,260 larvae were collected, with *An*. *funestus* group larvae accounting for 60.3% (n = 3,170) of the total, *Culex* spp 24.3 (n = 1,279), *An*. *gambiae s*.*l*. 8.3% (n = 438), and other anopheline larvae 7.1% (n = 373) (Table 1).

**Table 1:** Mean number and standard error (se) of different mosquito larvae species sampled from different aquatic habitats in the study areas.

Habitat information		Mean number and standard error (2se) of different mosquitoe larvae				
Habitat type	Total habitats	*An*. *funestus group*	*An*. *gambiae s*.*l*	Other anophelines	*Culex* spp	Total
Brick or sand pit	12	37.8(19.64)	2.7(2.5)	3.2(6.0)	15.8(11.9)	713
Ditch	8	16.5(8.4)	1.5(1.1)	5.0(7.4)	20.2(22.3)	346
Grounded pool	1	19.0(NA)	3.0(NA)	0	12.0(NA)	36
Man- made wells	20	11.2(3.4)	3.8(5.3)	0.6(0.6)	5.6(3. 6)	422
Rice field	2	40.5(51.9)	3.0(3.9)	10.5(20.6)	24.5(34.3)	157
River stream	34	61.7(29.8)	9.0(8.0)	5.4(4.7)	13.3(10.2)	3039
Spring- fed pool	2	25.0(17.6)	0	0	29.5(38.2)	109
Swamp	6	18.8(4.6)	0.7(1.0)	13.2(10.8)	40.7(19.4)	440
**Total**	85	3170	438	373	1279	5260

**Fig 2:**
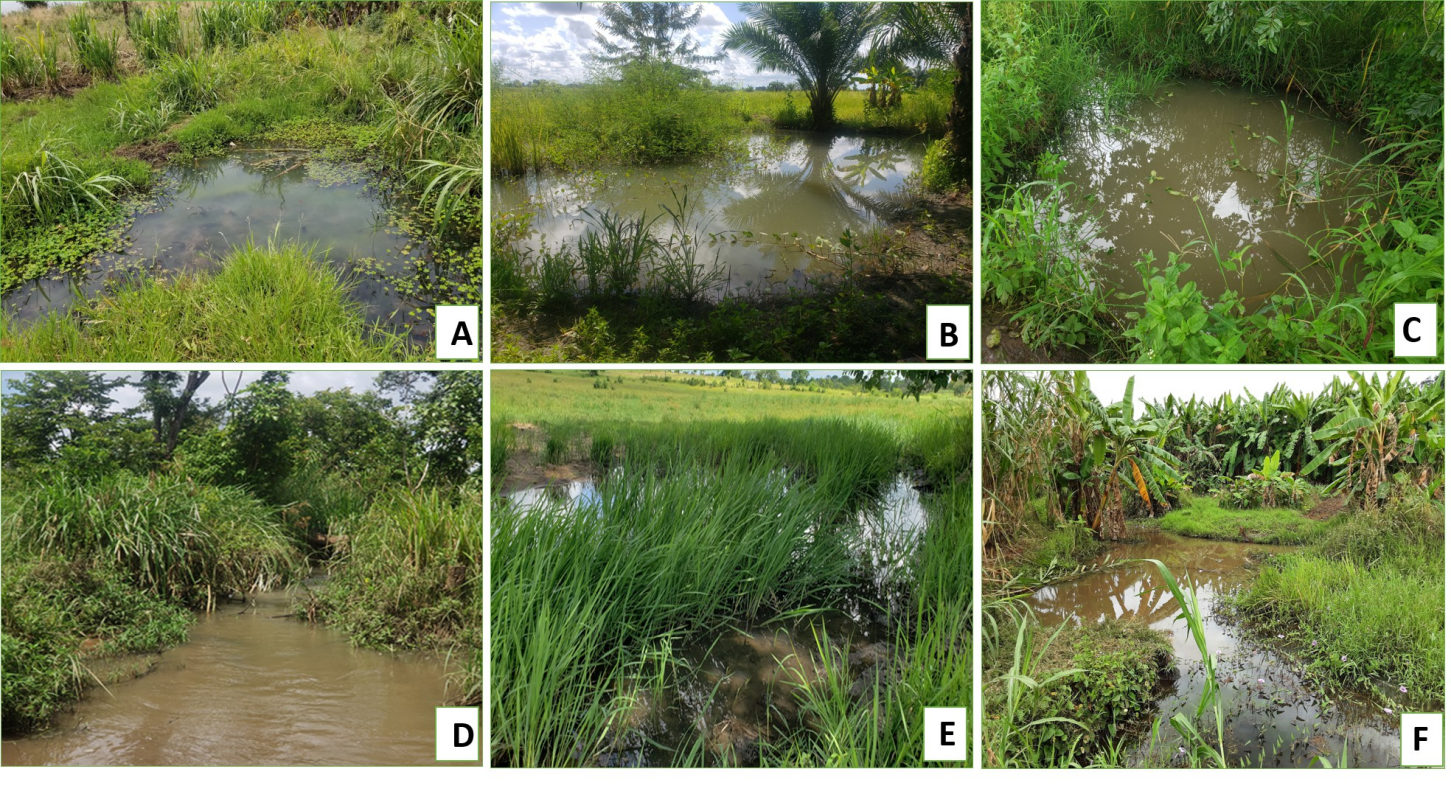
Different aquatic habitats which contain *Anopheles funestus* and predators. (**A**: Grounded pool, **B**: Brick/ sand pit, **C**: Man-made well, **D**: River stream, **E**: Swamp, **F**: Grounded fed pool.

Overall, *An*. *funestus* group larvae and predators were samples from different aquatic habitats both man made and natural habitats: includes grounded pool, Brick/ sand pit, man-made wells, river stream, swamp and spring fed pool (Fig 2). However, river stream, rice fields and brick or sand pit found to have higher mean number of *An*. *funestus* group larvae compared to all other habitats types (Table 1). River stream, spring fed pool and swamps found to have higher mean number of predators compared to all other habitats (Table 2).

**Table 2:** Mean number and standard error (se) of different predators sampled from different aquatic habitats in the study areas.

Habitat information		Mean number and standard error (2se) of different predators									
Habitat type	Total habitats (N)	Aeshnidae	Coenagrionidae	Dytiscidae	Notonectidae	Corixidae	Nepidae	Belomastidae	Amphibians	Unidentified species	Total
Brick or sand pit	12	8.25(6.07)	33.0(19.42)	3.83(2.61)	10.33(6.0)	19.42(22.95)	0.25(0.35)	1.42(1.59)	2.08(1.35)	2.0(2.84)	967
Ditch	8	1.0(1.11)	10.3(6.7)	7.63(7.27)	6.75(9.3)	1.13(0.94)	0.25(0.49)	3.25(5.58)	23.5(45.78)	2.25(1.92)	448
Grounded pool	1	0.0(0.0)	46.0(NA)	0.0(0.0)	0.0(0.0)	4.0(0.0)	0.0(0.0)	0.0(0.0)	1(NA)	0.0(0.0)	51
Man-made wells	20	7.05(6.38)	32.2(33.35)	2.45(2.53)	23.75(18.18)	5.95(6.89)	0.55(0.78)	0.05(0.10)	1.45(1.49)	0.90(0.98)	1487
Rice field	2	2.50(2.94)	37.5(42.14)	9.0(17.64)	5.0(9.8)	1.0(9.8)	1.00(1.96)	0.0(0.0)	1.00(0.00)	16.0(31.36)	129
River stream	34	1.50(0.98)	97.18(24.1)	2.0(1.5)	1.21(0.89)	17.4(11.0)	0.76(0.63)	1.65(1.33)	3.44(2.94)	6.76(4.06)	4485
Spring-fed pool	2	17.5(22.54)	46.5(46.07)	0.5(1.0)	36.5(2.94)	2.0(1.96)	1.00(0.0)	0.0(0.0)	2.0(1.96)	1.0(NA)	214
Swamp	6	11.0(7.62)	24.17(13.71)	11.7(12.0)	7.5(7.4)	16.17(15.48)	0.33(0.41)	0.50(0.98)	0.67(0.65)	10.83(4.54)	497
**Total**	85	405	4785	296	822	1060	48	103	370	389	8278

Generally, comparing to all other predator’s families, only six families namely Coenagrionidae, Corixidae, Notonectidae, Aeshnidae, Amphibians and Dytiscidae were more common and abundant in all aquatic habitats (Table 2).

### Characteristics of aquatic habitats and their influence on the abundance of *An*. *funestus* larval group and predators

*Anopheles funestus* group larvae and predators were found in high abundance in habitats larger than 100 m^2^ and at the edges of streams and rivers (habitats with fast moving water) (P<0.05, Tables 3 and 4), whilst low abundance of larvae was associated with habitats with submerged vegetation (P<0.05, Table 3). Predators were positively associated with the permanence of the aquatic habitats (P<0.005, Table 4). Other aquatic habitat characteristics including algae quantity and type, shade over the habitats, water depth, vegetation quantity, environment surrounding the aquatic habitats and the distance from the nearest houses were found to have no impact on *An*. *funestus* group larval abundance and predator abundance (Tables 3 and 4).

**Table 3:** Univariate and multivariate regression analysis of different aquatic habitat characteristics and their association with the abundance of *Anopheles funestus* larvae.

Aquatic habitat	Univariate analysis		Multivariate analysis	
	RR (95% LC, UC)	P-values	RR (95% LC, UC)	P-values
**Algae quantity**				
None	1		1	
Moderate	1.21 [0.66, 2.20]	0.535	1.44 [0.76, 2.73]	0.260
Abundant	1.94 [0.89, 4.20]	0.094	1.72 [0.72, 4.11]	0.224
**Habitat size**				
Less than 100 M	1		1	
Greater than 100M	2.54 [1.65, 3.89]	< 0.05	2.56 [1.58, 4.14]	< 0.05
**Vegetation type**				
None	1			
Emergent	0.73 [0.32, 1.63]	0.435	0.64 [0.35, 1.18]	0.151
Sub-merged	0.21 [0.04, 1.08]	0.062	0.10 [0.02, 0.57]	< 0.05
**Water Movement**				
Stagnant	1		1	
Slow	1.45 [0.90, 2.35]	0.127	1.29 [0.76, 2.18]	0.343
Fast	1.82 [0.96, 3.45]	0.064	2.79 [1.39, 5.63]	< 0.05
**Shade over habitat**				
None	1		1	
Partial	0.94 [0.55, 1.61]	0.818	1.13 [0.64, 2.00]	0.675
**Water depth**				
Less than 50 cm	1		1	
More than 50 cm	1.59 [1.07, 2.38]	0.02	0.94 [0.56, 1.59]	0.824
**Water type**				
Temporary	1		1	
Permanent	1.18 [0.76, 1.82]	0.456	0.77 [0.44, 1.33]	0.343
**Environment around habitat**				
Cultivated field	1		1	
Scrub	1.48 [0.94, 2.31]	0.089	1.30 [0.80, 2.11]	0. 282
**Water colour**				
Turbid	1		1	
Clear	0.99 [0.58, 1.71]	0.975	0.77 [0.46, 1.31]	0. 338
**Distance from houses**				
Less than 100	1			
More than 100 M	1.28 [0.79, 2.06]	0.320	1.02 [0.60, 1.75]	0.937

**Table 4:** Univariate and multivariate regression analysis of different aquatic habitat characteristics and their association with the abundance of predators.

Aquatic habitat	Univariate analysis		Multivariate analysis	
	RR (95% LC, UC)	P-values	RR (95% LC, UC)	P-values
**Habitat size**				
Less than 100 M	1		1	
Greater than 100M	2.92 [1.72, 4.96]	<0.05	3.52 [1.90, 6.53]	< 0.05
**Vegetation type**				
None	1			
Emergent	0.75 [0.37, 1.50]	0.409	1.17 [0.53, 2.56]	0.798
Submerged	0.42 [0.07, 2.54]	0.346	0.43 [0.08, 2.27]	0.323
**Water Movement**				
Stagnant	1		1	
Slow	2.09 [1.22, 3.58]	<0.05	0.98 [0.52, 1.87]	0.961
Fast	2.91[1.39, 6.13]	<0.05	2.25 [0.94, 5.39]	< 0.05
**Shade over habitat**				
None	1		1	
Partial	1.25 [0.64, 2.43]	0.508	1.66 [0.77, 3.56]	0.192
**Water depth**				
Less than 50 cm	1		1	
More than 50 cm	2.03 [1.17, 3.51]	<0.05	1.12 [0.58, 2.18]	0.727
**Water type**				
Temporary	1		1	
Permanent	2.64 [1.48, 4.69]	<0.05	2.89 [0.99, 4.41]	<0.05
**Environment around habitat**				
Cultivated field	1		1	
scrub	1.21 [0.71, 2.06]	0.477	1.06 [0.57, 1.98]	0.851
**Water colour**				
Coloured	1		1	
Clear	1.44 [0.56, 3.67]	0.447	0.86 [0.44, 1.68]	0.651
**Distance from home**				
Less than 100 M	1			
More than 100 M	1.164 [0.64, 2.12]	0.620	0.72 [0.35, 1.46]	0.364

### Water physicochemical parameters and their influence on the abundance of predators and *An*. *funestus* group larvae

There was no apparent association between physicochemical parameters (pH, temperature, TDS, EC and DO) and predator abundance (P>0.05, Table 5). Temperature, pH, TDS, EC also had no impact on the abundance of *An*. *funestus* group larvae but DO was positively associated with the abundance of *An*. *funestus* group larvae (P<0.05, Table 6).

**Table 5:** Univariate and multivariate analysis of associations between water physicochemical parameters and the abundance of predators in *Anopheles funestus* aquatic habitats.

		Univariate analysis Multivariate analysis			
Water characteristics	Mean (Range)	RR (95% LC, UC)	P-values	RR (95% LC, UC)	P-values
pH	6.3 [5.70–7.82]	1.00 [0.74, 1.39]	0.981	1.17 [0.84, 1.64]	0.356
Temperature(^0^C)	28.0 [23.3–36.]	0.85 [0.59, 1.23]	0.390	0.84 [0.57, 1.24]	0.381
TDS (ppm)	126.8[23.0–395.0]	1.31 [0.99, 1.74]	0.056	1.44 [0.98, 2.13]	0.064
EC (μS/cm)	253.7 [40.1–619.0]	1.17 [0.86, 1.58]	0.312	0.83 [0.53, 1.31]	0.432
DO (mg/L)	6.2[1.12–16.56]	1.25 [0.93, 1.68]	0.142	1.31 [0.93, 1.85]	1.118

**Table 6:** Univariate and multivariate analysis of associations between water physicochemical parameters and the abundance of *Anopheles funestus* larvae.

		Univariate analysis		Multivariate analysis	
Water characteristics	Mean (Range)	RR (95% LC, UC)	P-values	RR (95% LC, UC)	P-values
pH	6.3 [5.70–7.82]	0.92 [0.67,1.26]	0.620	0.91 [0.68,1.23]	0.542
Temperature(^0^C)	28.0 [23.3–36.]	0.89 [0.70, 1.14]	0.372	0.86 [0.68, 1.10]	0.231
TDS (ppm)	126.8 [23.0–395.0]	1.01 [0.74, 1.36]	0.910	1.01 [0.72, 1.42]	0.956
EC (μS/cm)	253.7 [40.1–619.0]	1.05 [0.77, 1.43]	0.770	0.96 [0.66, 1.40]	0.811
DO (mg/L)	6.2 [1.12–16.56]	1.43 [1.08, 1.88]	0.010	1.47 [1.11, 1.94]	< 0.05

### Association of different predators with the abundance of *An*. *funestus* group larvae

Coenagrionidae and Dytiscidae were positively associated with *An*. *funestus* group larval abundance (P<0.05) whilst Notonectidae and Corixidae were negatively associated with *An*. *funestus* abundance (P<0.05). No strong association between abundance of *An*. *funestus* group larval abundance and some predator families including Aeshnidae and Belostomatidae were found (P>0.05) (Table 7).

**Table 7:** Univariate and multivariate regression analysis of different predators and their association with the abundance of *Anopheles funestus* larvae in the aquatic habitats.

Aquatic predators	Univariate analysis		Multivariate analysis	
	RR (95% LC, UC)	P-values	RR (95% LC, UC)	P-value
**Aeshnidae**	1.007[0.984, 1.031]	0.524	1.014 [0.989,1.039]	0.268
**Coenagrionidae**	1.008 [1.005, 1.011]	<0.05	1.008 [1.006,1.011]	<0.05
**Dytiscidae**	1.043 [1.007, 1.078]	<0.05	1.035 [1.006,1.065]	<0.05
**Notonectidae**	0.998 [0.990, 1.006]	0.685	0.988 [0.978,0.998]	<0.05
**Corixidae**	0.994 [0.984, 1.005]	0.274	0.990 [0,984,0.997]	<0.05
**Belomastidae**	0.99 [0.921, 1.069]	0.794	0.973 [0.920,1.028]	0.327

### Co-existence of different mosquito species and different predator families in the aquatic habitats

Among 85 *An*. *funestus* habitats, a total of 46 were co-inhabited by *An*. *gambiae s*.*l*, 23 habitats had other anopheline larvae and 60 habitats had *Culex* spp (S1 Table). Furthermore, Coenagrionidae were found in 75 habitats, Aeshnidae in 48 habitats, Corixidae in 49 habitats, Notonectidae in 44 habitats, Dytiscidae in 37 habitats, Nepidae in 21 habitats, Amphibian in 33 habitats, Belostomatidae in 18 habitats and unidentified group in 42 habitats (S2 Table).

## Discussion

Ecological interactions such as predation and competition are key drivers of population size of numerous organisms [31]. In the context of mosquito borne diseases, predators play an important role in regulating the diseases transmitting mosquitoes directly through feeding on mosquito larvae or indirectly through compromising mosquito fecundity, growth rate and growth trajectories [13, 31]. Also, they regulate the *Anopheles* populations naturally through predation, parasitism and competition [32], the use of aquatic predators represents a potentially simple and practical biological technology for the control of disease transmitting mosquitoes [8]. Biological control methods, including the use of naturally occurring predators, have been utilised for vector control in many parts of the world [14, 33, 34].

The present study was undertaken to investigate the common predators and factors influencing their abundance in *An*. *funestus* aquatic habitats. Eight different families of predators co-existing with *An*. *funestus* group were identified, six of which, namely; Coenagrionidae, Corixidae, Notonectidae, Aeshnidae, Amphibians, and Dytiscidae were common in all habitat types. Similarly, previous studies confirmed different predominant family Coenagrionidae [35], Dytiscidae [35], Notonectidae [36] in mosquitoes larval habitats. For example Gilbert and Burns, found that Notonectidae have direct effects on mosquito larvae population [37]. Current study found consistently, high mean number of Coenagrionidae family in all habitats type as compare to other predator families. This implies that the characteristics of the surveyed aquatic habitats were favouring the survival and growth of these predators, hence led to high abundance.

Variation and abundance of different predators across different aquatic habitats were strongly associated with some physical characteristics of the habitat. The high abundance of predators were generally observed in permanent aquatic habitats with fast moving water and larger than 100m^2^, e.g. brick or sand pits, man-made wells, river streams and swamps, similar to observations from other settings [16, 38–42]. Such permanent aquatic habitats contain favourable amounts of both decomposed organic and inorganic matter which serve as food for predators, and these habitats allow colonization of the predators than temporal and simple structural habitats [43].

Interestingly, it was noted that aquatic habitats larger than 100m^2^ with fast moving water were positively associated with the abundance of *An*. *funestus* group larvae. Aquatic habitats with submerged vegetation were negatively associated with the abundance of *An*. *funestus* group larvae. Previous studies have described a positive association between aquatic habitats with emergent vegetation and abundance of *An*. *funestus* group larvae [24, 44], but not a negative association between abundance and habitats with submerged vegetation. This may be due to the season in which sampling was undertaken may have an influence on the nature of the vegetation in the aquatic habitats and movement of water.

With the exception of DO, there was no statistically significant association between other water physicochemical parameters and the abundance of *An*. *funestus* group larvae. On the other hand, predator abundance was not significantly impacted by any of the measured water physicochemical parameters. In contrary to the previous study, dissolved oxygen was found to be positively associated with the abundance of *An*. *funestus* group larvae [45]. This may be due to the preference of *An*. *funestus* larvae to breed in fresh and clear water which contains high levels of dissolved oxygen.

The current results are in line with the findings reported by *Bashar et al*., [46], which indicated that dissolved oxygen is the preeminent predictor for the abundance of *Anopheles* mosquito larvae in aquatic habitat. Several factors, such as physical, chemical, biological and microbiological processes influence the levels of dissolved oxygen concentration in water, such that low dissolved oxygen concentrations, < 3 mg/L in fresh water indicate high level of pollution [35]. In this study the mean of dissolved oxygen was 6.2 mg/L and ranges from 1.12–16.56 mg/L, this indicates that most of these aquatic habitats contained the highest amount of dissolved oxygen and aeration which favoured the abundance of the *An*. *funestus* group larvae and predators.

Water pH is one of the important factors for aquatic organisms [47]. It can limit the abundance and distribution of aquatic organisms because it is directly related to their cellular functions [47], growth and development as well as their survival [47, 48]. It has been noted that mosquitoes can tolerate extremely high levels of water pH [47, 48]. However, the level of pH tolerance can be associated with the abundance of these species present in the environment [47]. The current study shows that pH was not statistically significant associated with the abundance of either *An*. *funestus* group larvae or predators in the aquatic habitats. This correlates with Akeju *et al*., [49], Obi *et al*. [50] and Chaiphongpachara *et al*., [35, 51] and suggests that *An*. *funestus* group larvae and predators are able to tolerate a wide range of pH in different environments. In addition, the current study shows the range of pH in the aquatic habitats was 5.70 to 7.82. These results are in line with the previous findings which shows the association of *Anopheles* larvae with aquatic insects including predators in a wide range of pH concentration in their aquatic habitats [35, 52]. Both mosquito larvae and aquatic insects including predators have the mechanisms that enable them to inhabit such environments [47].

Temperature is an important factor mediating predators and mosquito larvae interactions [53]. For example, it affects the ecology, physiology, metabolic processes and overall fitness of organisms [54]. Implication on the interaction between predators and mosquitoes as well as their behaviour performance in the aquatic habitats is mediated by temperature, because temperature plays an essential role as a regulatory mechanism that drives both physiological and biochemical activities [55]. Both *An*. *funestus* group larvae and predators share the same aquatic habitats which their temperature ranged from 23.3–36.4°C, this shows that *An*. *funestus* and predators preferred warm conditions for their survival, development and colonization. The findings of the current study are in line with findings by Dida *et al*., which reported that both predators and prey preferred temperatures above 18°C and above 25°C [35].

Temperature was not statistically significant associated with the abundance of *An*. *funestus* group larvae and predators in the aquatic habitats which correlates previous findings [24], however some studies reported contrary findings showing positive association between *An*. *funestus* larvae and by temperature [45, 46, 49, 56]. Most studies have mainly focused on the impacts of terrestrial temperature on mosquitoes but a limited number of studies focussed on aquatic habitats in the context of thermal tolerance, particularly for vector mosquitoes and their predators. This necessitates further investigations across seasons.

While studies done elsewhere yielded evidence that electrical conductivity is positively associated with the abundance of *An*. *funestus* larvae [49, 56]. Further study found that higher levels of electrical conductivity was due to the application of agricultural fertilisers, pesticides and herbicides [57], but this study did not find any significant association between electrical conductivity and abundance of both *An*. *funestus* group larvae and predators in the aquatic habitats. Electrical conductivity ranged between 40.1–619.0 μS/cm, this shows *An*. *funestus* group larvae and predators can survive in a wide range of electrical conductivity in the aquatic habitats which similar to report by *Dida et al*., which suggested mosquito larvae and predators were most preferable in the aquatic habitats with electrical conductivity ranges between 162.9μS/cm and 166 μS/cm [35].

In aquatic habitats, higher total dissolved solids have harmful impacts on the aquatic organisms [58]. It changes the mineral water contents, which is important for survival of predators and mosquito larvae [58]. Furthermore, it determines the flow of water out of an organism’s cell. In this study, there was a wide range of total dissolved solids in the aquatic habitats in which it ranges between 23.0–395.0 ppm. This variation might be the same as previously reported by another study that total dissolved solids in the aquatic habitat is highly dependent on the different factors such as the pattern use of different chemicals in the environments (like agriculture pesticides) [59]. Also, these results correlates to Oyewole *et al*., [60], but contrary to Abai *et al*., and Dida *et al*., which suggested that *Anopheles* mosquito associated with very high total dissolved solids (1,261.40 ± 1,214.31) [61] or very low 8–87 ppm [35].

This study revealed that various predator families share similar aquatic habitats. Furthermore, it found that *An*. *funestus* group larvae coexist with various mosquito species and other organisms in their aquatic habitats, which is consistent with previous studies conducted by Nambunga *et al*., [24] and Dida *et al*., [35].

The association of *An*. *funestus* group larvae and predators was varying. In particular, this study noted some predator families namely; Notonectidae and Corixidae were bounded in area with low abundance of *An*. *funestus* group larvae. However, the highest number of predators and low number of mosquito larvae could also reflect the direct predation in the aquatic habitats. Whilst Coenagrionidae and Dytiscidae were bounded in the area with higher abundance of *An*. *funestus* group larvae, showing the positive association between these predators and *An*. *funestus* group larvae. These could be due to variation in feeding preferences among each predator family. More important, further studies should be done to confirm this because another study has shown that Coenagrionidae are not only significant predators for *Anopheles* larvae but also *Aedes aegypti* larvae [38]. However, the current study did not find a clear and significant association between different predator families like Aeshndae and Belostomatidae with *An*. *funestus* group larvae.

This study suggests that, for effective malaria vector control, intervention strategies should focus on both, permanent aquatic habitats and temporary/seasonal as well as micro-habitats such as ditches and some man-made wells. The evidence by this study also suggests that these temporary and micro habitats can significantly produce higher numbers of disease transmitting mosquitoes though they limit predator’s colonization abilities. One benefit of utilising biological control is that it may target mosquito species at low densities, it has no impact on non-target organisms and it is simple to use in the field [32].

One of the limitations of this study was that it did not focus on understanding anthropogenic factors and how they might influence the abundance of predators. Though it is very important to assess how both natural and human activities influence abundance of predators, the current study focused on water physicochemical parameters and other physical characteristics of the aquatic habitats. Further studies should also, morphologically identify the aquatic predators to species level using an appropriate identification key. This should help to understand how the aquatic predators are distributed in different aquatic habitats. Although this does not affect our interpretation of results, a cross sectional study doesn’t represent the variation among habitat characteristics over time including the changes in temperature and water physicochemical parameters. A longitudinal study would help capture seasonal variations between predator and prey abundance. Such study may help in the design of novel interventions focussed on this relationship.

## Conclusion

This study demonstrated the existence of common predators in aquatic habitats colonized by *An*. *funestus* group larvae and factors influencing their abundance. Six predator families were commonly identified; Coenagrionidae, Corixidae, Notonectidae, Amphibians, Aeshndae, and Dytiscidae. The abundance of predator families with *An*. *funestus* group larvae varied. The only physicochemical parameter influencing *An*. *funestus* group larvae abundance was dissolved oxygen. Additional studies are needed to demonstrate the efficacy of predators on mosquito larval densities and adult fitness traits. Interventions leveraging the interaction between mosquitoes and predators can be established to disrupt the transmission potential and survival of the *An*. *funestus* mosquitoes.

## Supporting information

S1 Table Number of each aquatic habitats type showing the co-existence of different mosquito group larvae. (DOCX)

S2 Table Number of aquatic habitats type showing the co-existence of different predators. (DOCX)

S1 Dataset Common predators. (CSV)

## List of abbreviations

DO: Dissolved oxygen
EC: Electrical conductivity
ITNs: Insecticide Treated Nets
IRS: Indoor Residual Spraying
LLINs: Long lasting Insecticides Treated Nets
TDS: Total dissolved solids
WHO: World Health Organization
