# Supplementary material for: Common predators and factors influencing their abundance in Anopheles funestus aquatic habitats in rural south-eastern Tanzania
Source: PLoS One. 2023 Jun 26;18(6):e0287655. doi: 10.1371/journal.pone.0287655 (PMC10292713; doi:10.1371/journal.pone.0287655)
Supplement: S2 Table — (DOCX) [file pone.0287655.s002.docx]

**Common predators and factors influencing their abundance in *Anopheles funestus* aquatic habitats in rural south-eastern Tanzania**

Herieth H. Mahenge^1, 2*^_,_ Letus L. Muyaga^1^, Joel D. Nkya^1,^ Khamis S. Kifungo^1^ Najat F. Kahamba^1, 3^, Halfan S. Ngowo^1,3¶^, Emmanuel W. Kaindoa^1,2,4¶^

1. Environmental Health and Ecological Sciences Department, Ifakara Health Institute, P. O. Box 53, Ifakara, Tanzania.
2. The Nelson Mandela, African Institution of Science and Technology, School of Life Sciences and Bio Engineering, Tengeru, Arusha, United Republic of Tanzania.
3. School of Biodervisty, Animal Health and Comparative Medicine, G12 8QQ, University of Glasgow, UK.
4. Wits Research Institute for Malaria, School of Pathology, Faculty of Health Sciences, University of the Witwatersrand and the Centre for Emerging Zoonotic and Parasitic Diseases, National Institute for Communicable Diseases, Johannesburg, South Africa.

*Corresponding author: [hmahenge@ihi.or.tz](mailto:hmahenge@ihi.or.tz)

¶Contributed equally

**Email address:**

1. Letus L. Muyaga: [lmuyaga@ihi.or.tz](mailto:lmuyaga@ihi.or.tz)
2. Joel D. Nkya: joelnkya20@gmail.com
3. Khamis S. Kifungo: [hkifungo@ihi.or.tz](mailto:hkifungo@ihi.or.tz)
4. Najat F. Kahamba: [nkahamba@ihi.or.tz](mailto:nkahamba@ihi.or.tz)
5. Halfan S. Ngowo: [hngowo@ihi.or.tz](mailto:hngowo@ihi.or.tz)
6. Emmanuel W. Kaindoa: [ekaindoa@ihi.or.tz](mailto:ekaindoa@ihi.or.tz)

Table S2. Number of aquatic habitats type showing the co-existence of different predators.

| **Habitat information** | | **Number of habitats with different predators** | | | | | | | | |
| --- | --- | --- | --- | --- | --- | --- | --- | --- | --- | --- |
| **Habitat type** | **Number of**  **habitat** | **Aeshnidae** | **Coenagrionidae-** | **Dytiscidae** | **Notonectidae** | **Corixidae** | **Nepidae** | **Belostomatidae** | **Amphibians** | **Unidentified group** |
| Brick or sand pit | 12 | 10 | 12 | 8 | 8 | 7 | 2 | 4 | 6 | 3 |
| Ditch | 8 | 3 | 5 | 5 | 4 | 5 | 1 | 2 | 2 | 4 |
| Grounded pool | 1 | 0 | 1 | 0 | 0 | 1 | 0 | 0 | 1 | 0 |
| Man-made wells | 20 | 12 | 14 | 6 | 14 | 7 | 4 | 1 | 6 | 5 |
| Rice field | 2 | 2 | 2 | 1 | 1 | 2 | 1 | 0 | 2 | 1 |
| River stream | 34 | 13 | 34 | 11 | 10 | 21 | 9 | 10 | 11 | 21 |
| Spring-fed pool | 2 | 2 | 2 | 1 | 2 | 2 | 2 | 0 | 2 | 2 |
| Swamp | 6 | 6 | 6 | 5 | 5 | 4 | 2 | 1 | 3 | 6 |
| **Total** | **85** | **48** | **75** | **37** | **44** | **49** | **21** | **18** | **33** | **42** |
